# Epigenetic Enhancement of the Post-replicative DNA Mismatch Repair of Mammalian Genomes by a Hemi-^m^CpG-Np95-Dnmt1 Axis

**DOI:** 10.1038/srep37490

**Published:** 2016-11-25

**Authors:** Keh-Yang Wang, Chun-Chang Chen, Shih-Feng Tsai, Che-Kun James Shen

**Affiliations:** 1 Institute of Molecular Biology, Academia Sinica, Nankang, Taipei 11529, Taiwan; 2 Genome Research Center, National Yang-Ming University, Taipei 11221, Taiwan; 3 Department of Life Sciences and Institute of Genome Sciences, National Yang-Ming University, Taipei 11221, Taiwan; 4 Institute of Molecular and Genomic Medicine, National Health Research Institutes, Miaoli 35053, Taiwan

## Abstract

DNA methylation at C of CpG dyads (^m^CpG) in vertebrate genomes is essential for gene regulation, genome stability and development. We show in this study that proper functioning of post-replicative DNA mismatch repair (MMR) in mammalian cells relies on the presence of genomic ^m^CpG, as well as on the maintenance DNA methyltransferase Dnmt1 independently of its catalytic activity. More importantly, high efficiency of mammalian MMR surveillance is achieved through a hemi-^m^CpG-Np95(Uhrf1)-Dnmt1 axis, in which the MMR surveillance complex(es) is recruited to post-replicative DNA by Dnmt1, requiring its interactions with MutSα, as well as with Np95 bound at the hemi-methylated CpG sites. Thus, efficiency of MMR surveillance over the mammalian genome *in vivo* is enhanced at the epigenetic level. This synergy endows vertebrate CpG methylation with a new biological significance and, consequently, an additional mechanism for the maintenance of vertebrate genome stability.

MMR ensures genomic stability by correcting DNA mispairs generated during DNA biosynthesis or exposure to genotoxic methylating agents^1^. Defects in MMR are manifested by microsatellite instability (MSI)^2^ and higher resistance to DNA alkylation^3^,^4^. The best characterized MMR is the methyl-directed MMR in *E. coli* that bears G^m^ATC sequences methylated by DNA-(N6-adenine) methyltransferase (Dam). In *E. coli*, mismatch recognition, i.e. the initial step of MMR, is mediated by the homodimeric MutS proteins. The MutL homodimer then complexes with the mismatch-bound MutS, which recruits the site-specific endonuclease MutH that nicks the unmethylated nascent strand of the hemi-methylated GATC sequence just 5′ to the G. This mismatch-provoked and GATC-Dam-MutH-mediated nick generation provides the strand-specificity of MMR in *E. coli*, as subsequent excision of the nascent DNA strand can initiate from the nick of either side of the mismatch and terminate several bases beyond the mispair. Finally, MMR is completed by repair DNA synthesis and ligation^4^,^5^,^6^.

The general MMR system in eukaryotic cells is dependent on eukaryotic homologs of the bacterial *mutS* and *mutL* gene products. The major eukaryotic players in recognition of DNA mismatches are MutSα and MutSβ. MutSα is a heterodimer of MSH2 and MSH6, and it appears to be the predominant mismatch-binding factor in human cells, while MutSβ is an MSH2-MSH3 heterodimer^1^,^7^. As for the *E. coli* MutS homologs, eukaryotic MutL homologs consist of three heterodimers, including MutLα (MLH1-PMS2), MutLβ (MLH1-PMS1) and MutLγ (MLH1-MLH3) in mammalian cells. Similar to the bacterial MutL homodimer, MutLα can interact with either MutSα or MutSβ and constitutes the primary MutL activity for eukaryotic mismatch correction^7^. However, MutLβ and MutLγ only play minor roles in MMR by repairing a subset of insertion/deletion loops^1^,^7^,^8^. In contrast to the *E. coli* system, the eukaryotic MMR system lacks DNA Dam methylation and the MutH homologs^1^. However, similar to *E. coli*, the eukaryotic MMR machinery is an integral part of the large replication factory. Several DNA replication factors, including the proliferating cell nuclear antigen (PCNA), have been implicated in eukaryotic MMR^4^. In particular, activation of the latent endonuclease activity of MutLα^9^,^10^ through physical interaction with PCNA *in vitro* implies that repair specificity on the newly synthesized strand could also be directed by the loading orientation of PCNA at the replication fork^11^, with the resulting nicks being suggested as entry sites for the downstream repair actions of the eukaryotic MMR *in vivo*.

Faithful replication not only of genetic information but also epigenetic information is indispensable in inheritance. In vertebrates, DNA methylation occurs primarily on cytosine in CpG dyads, and their genomic methylation patterns are established and maintained by the DNA (cytosine-5)-methyltransferases, or Dnmts. Among them, Dnmt3a and Dnmt3b are responsible for *de novo* methylation of the genome, whereas Dnmt1 maintains the methylation patterns following DNA replication. During replication, Dnmt1 is localized at the replication foci, in part through its interaction with PCNA^12^,^13^, to maintain methylation patterns. Interestingly, Np95, also known as Uhrf1 and ICBP90, has been identified as a hemi-^m^CpG-binding factor that plays an essential role in the recruitment of Dnmt1 for performance of post-replicative maintenance methylation of the vertebrate genome^14^,^15^. Loss of Dnmt1 results in DNA hypomethylation and lethality during mouse development^16^. However, in contrast to the essential role of Dnmt1 for survival of the differentiated cells^17^,^18^, mouse embryonic stem (ES) cells can proliferate normally with complete inactivation of Dnmt1 or the whole DNA methylation system^19^,^20^, which provides an opportunity for studying the function of DNA methylation system independently of its essential role in transcriptional regulation.

A link between MMR and Dnmt1 in vertebrate cells has been identified through different genetic approaches. Dnmt1-deficient ES cells generated by three independent methods all exhibited MMR defects manifested by MSI of either the endogenous alleles^21^ or the exogenous sequences^22^,^23^, or by a tolerance to 6-thioguanine (6-TG) treatment^22^. Similarly, contraction of the CAG repeat number in a reporter gene was observed in CHO cells treated with hydralazine to inhibit the expression of Dnmt1^24^. Despite these results, however, the molecular basis of how Dnmt1 participates in MMR of vertebrate genomes has remained unclear.

Here, we show by genetic approaches that proper post-replicative MMR is maintained by a sufficiently high level of Dnmt1 protein, independently of its catalytic activity and interaction with PCNA *in vivo*. Furthermore, this function of Dnmt1 in MMR is mediated through its Np95-dependent distribution on the chromatin, which in turn aids the chromatin loading of the MMR proteins through a hemi-^m^CpG-Np95-Dnmt1 axis, in which the MMR surveillance complex(es) is recruited to post-replicative DNA by Dnmt1, requiring its interactions with MutSα as well as with Np95 bound at the hemi-methylated CpG sites. Thus, efficiency of MMR surveillance over the mammalian genome is enhanced at the epigenetic level in an *in vivo* context.

## Results

### Establishment and characterization of stable ES clones with ectopic expression of Dnmt1 or its variants in a *Dnmt1* ^*−/−*^ ES cell line

**Figure 1:**
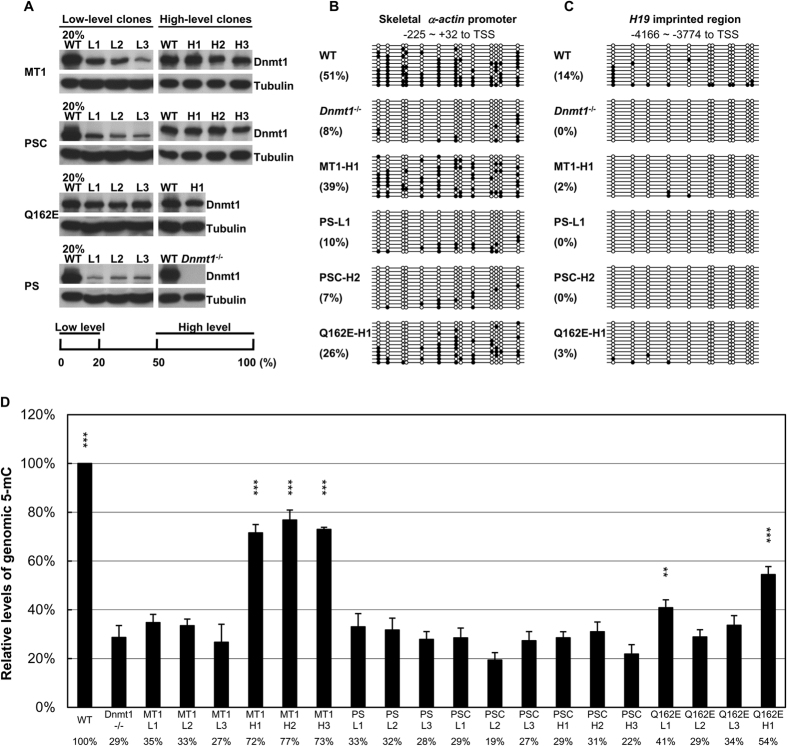
Establishment and characterization of stable ES clones with ectopic expression of Dnmt1 or its variants in a *Dnmt1*^*−/−*^ ES cell line. (**A**) Expression levels of Dnmt1 and its variants in the stable ES clones. There was no Dnmt1 signal from *Dnmt1*^*−/−*^ cells. Signal from the Dnmt1/variants in the low-level clones (L1~L3) was less than that from endogenous Dnmt1 in the wild-type ES cells (WT), in which only 20% as much lysate was loaded (the left four panels) and the high-level clones (H1~H3) expressed 50% or more of the Dnmt1/variants than the endogenous Dnmt1 in WT (the right three panels). For comparison, all the Dnmt1/variant signals on the Western blots are normalized against tubulin. (**B,C**) DNA methylation patterns of specific genomic regions. Bisulfite sequencing was conducted to examine the methylation status of the skeletal *α–actin* promoter (**B**) and imprinted *H19* gene (**C**). Each line represents a single DNA methylation profiling result of bisulfite sequencing and twelve profiles of each genomic DNA sample were used to calculate the percentage of 5-mC in the CpG sites, as shown in the parentheses. ○, un-methylated CpG; **•**, methylated CpG; TSS, transcription start site. (**D**) HPLC analysis of the genomic 5-mC levels. The genomic contents of 5-mC of the different ES clones are shown in the histobar diagram as the percentages relative to the WT. Each mean ± SD was derived from three independent DNA preparations and measurements. The percentage of the mean is shown below each bar and compared to that of the *Dnmt1*^*−/−*^ cells. Student’s *t* test; **p < 0.01; ***p < 0.001.

To dissect the regulatory mechanisms of the vertebrate MMR by Dnmt1, *Dnmt1*^*−/−*^ ES cells were stably transfected with plasmids expressing wild-type *Dnmt1* minigene (MT1) or its variants. The Dnmt1 variants included methylation-inactive forms, PSC^25^ and PS^26^, and the Q162E variant^27^ with a reduced affinity to bind PCNA. Stable clones were selected and the levels of the exogenous proteins were estimated by Western blotting (Fig. 1A). Interestingly, levels as low as 10% of normal Dnmt1 amount could sustain mouse development^28^, which prompted us to collect ES clones with different levels of the exogenous proteins for study. Most of the stable clones expressed less exogenous Dnmt1 or its variants than the wild-type (WT) ES cells expressed endogenous Dnmt1. We categorized those clones with expression of exogenous Dnmt1 or variants less than 20% of the Dnmt1 expression in WT ES cells as “low-level” clones (Fig. 1A, L1~L3). Note that the Dnmt1/variant signals of “low-level” clones were compared to the Dnmt1 signal from 20% of the WT sample loaded on the same gel/blot. In contrast, fewer clones with exogenous expression of Dnmt1 or variants higher than 50% of Dnmt1 in the WT ES cells were obtained, and they were categorized as “high-level” clones (Fig. 1A, H1~H3). Three independent “low-level” clones and “high-level” clones expressing the MT1 or the PSC variant, respectively, were randomly picked for further analyses. However, only one “high-level” clone expressing the Q162E variant could be obtained and no “high-level” clone expressing the PS variant was available (Fig. 1A).

In addition to their expression levels, the enzymatic activities of the ectopically expressed Dnmt1 or its variants were validated by checking the methylation status of the genomic DNAs from the different stable clones using bisulfite sequencing of the skeletal *α–actin* promoter and imprinted *H19* gene. As exemplified in Fig. 1B and C, 51% and 14% of the CpG sites were methylated in the skeletal *α–actin* promoter and *H19* region, respectively, in the WT ES cells. As expected, the ^m^CpG percentages of the two regions were only 8% and zero, respectively, in *Dnmt1*^*−/−*^ ES cells (Fig. 1B,C). Among the stable ES clones analyzed, re-methylation of the skeletal *α–actin* promoter occurred in the MT1-H1 (39%) and Q162E-H1 (26%) clones. In contrast, neither PS (PS-L1) nor a high level of PSC (PSC-H2) showed re-methylation of the skeletal *α–actin* promoter (Fig. 1B). The methylation pattern of the *H19* region was not recovered in any of the stable ES clones analyzed (Fig. 1C), since germ-line passage was required for re-establishment of the methylation status of imprinted genes^29^.

We also measured the genomic 5-methylcytosine (5-mC) contents of the different ES clones by HPLC. Remnant amounts of 5-mC in *Dnmt1*^*−/−*^ cells were reduced to 29% that of the WT cells (Fig. 1D). While little change of DNA methylation status occurred in ES clones expressing low levels of MT1 or Q162E, the 5-mC levels were increased significantly in ES clones expressing high levels of MT1 or Q162E (Fig. 1D). As expected, exogenous expression of the PS or PSC variant, whether in high or low amounts, had little effect on the genomic 5-mC content (Fig. 1D). The bisulfite sequencing and HPLC analyses together validated the correlation between the extents of genomic DNA methylation and the presence of catalytically active/inactive Dnmt1 proteins in the various WT, *Dnmt1*^*−/−*^, and stable ES clones.

### Rescue of MMR by ectopically expressed Dnmt1 and its variants

#### Assay of 6-TG sensitivity

**Figure 2:**
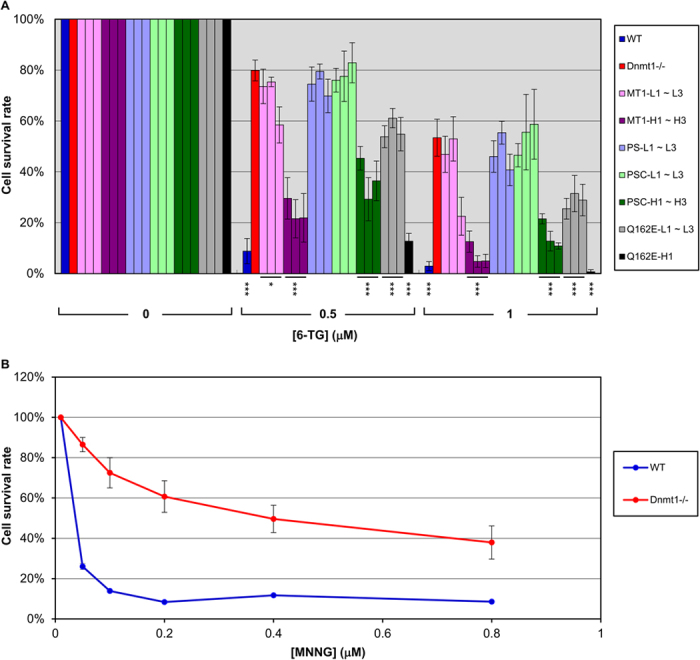
Expression level-dependent rescue of MMR deficiency in a *Dnmt1*^*−/−*^ ES cell line by ectopic expression of Dnmt1 or its variants. (**A**) The MMR function in the stable ES clones was evaluated by 6-TG sensitivity assays. The cell survival rates derived from three sets of duplicated experiments are presented in the histobar diagram as mean ± SEM. Bars in different colors are used for the individual ES clones expressing different exogenous Dnmt1/variants. The clonal order, e.g. MT1-L1, MT1-L2, MT1-L3, is presented from left to right. Most strikingly, in contrast to the low levels of the inactive Dnmt1 variants PS (PS-L1~L3, the light blue bars) and PSC (PSC-L1~L3, the light green bars), exogenous expression of high levels of the PSC variant of Dnmt1 (PSC-H1~H3, the dark green bars) restored 6-TG sensitivity to an extent comparable to ES clones expressing high levels of the active Dnmt1 (MT1-H1~H3, the purple bars and Q162E-H1, the black bars). The significances were calculated by comparing with the average survival rate of the *Dnmt1*^*−/−*^ cells (red bars). Student’s *t* test, *p < 0.05; ***p < 0.001. (**B**) Resistance of *Dnmt1*^*−/−*^ ES cells to MNNG. The WT and *Dnmt1*^*−/−*^ ES cell lines were treated with increasing amounts of MNNG in the presence of O^6^-benzylguanine. The curves of cell survival rates derived from four sets of duplicated experiments are presented as mean ± SEM.

6-TG incorporated into DNA is modified and recognized as a mismatch lesion, triggering futile repair cycles of MMR^3^. Since the cytotoxicity of 6-TG is mediated by MMR, cells with MMR deficiencies would be expected to be more resistant to the drug. As shown in Fig. 2A, all of the ES clones exhibited dosage-dependent sensitivities to 6-TG treatment. However, *Dnmt1*^*−/−*^ cells (Fig. 2A, red bars) were significantly more resistant to 6-TG than the WT cells (Fig. 2A, dark blue bars), consistent with the previous finding of MMR deficiency in *Dnmt1*^*−/−*^ cells^22^. Interestingly, while low-level expression of exogenous Dnmt1 or Q162E (Fig. 2A, pink bars and grey bars) could rescue a small amount of the 6-TG sensitivity, high level expression of either one greatly restored the 6-TG sensitivity of the ES cells (Fig. 2A, purple bars and black bars). This result suggests that Dnmt1 plays a positive role in MMR and that the point mutation Q162E affecting the binding affinity of Dnmt1 with PCNA did not alter its function in MMR.

Surprisingly though, in contrast to the low levels of the inactive Dnmt1 variants PS (Fig. 2A, light blue bars) and PSC (Fig. 2A, light green bars), the methylation-inactive PSC variant recaptured the 6-TG sensitive phenotype in all three “high-level” clones (Fig. 2A, dark green bars). Collectively, the data of Fig. 2A demonstrate that a sufficient cellular level of Dnmt1 is required for its function in MMR, but independently of its catalytic activity and PCNA-binding capability.

Since metabolic activation of 6-TG was a prerequisite of its incorporation into the genome during DNA replication, we also evaluated the effect of Dnmt1 deficiency on MMR by comparing the sensitivities of WT and *Dnmt1*^*−/−*^ ES cells to another DNA methylating agent, i.e. N-methyl-N′-nitro-N-nitrosoguanidine (MNNG). MNNG modifies guanine on DNA to generate O^6^-methylguanine, which can be repaired through O^6^-methylguanine DNA methyltransferase by directly removing the methyl group. When O^6^-benzylguanine was included in growth medium, repair activity of O^6^-methylguanine DNA methyltransferase was competitively inhibited and the MNNG-induced O^6^-methylguanine on the genome provoked MMR-dependent cytotoxicity^4^,^30^. As shown in Fig. 2B, the median lethal dose of MNNG for *Dnmt1*^*−/−*^ ES cells increased ~12-fold compared to WT ES cells. Thus, the MNNG resistance phenotype of *Dnmt1*^*−/−*^ ES cells observed in Fig. 2B reconfirmed that the 6-TG sensitivities of the different *Dnmt1*^*−/−*^ ES clones described in Fig. 2A and reported in a previous study^22^ were due to deficiency of MMR rather than disturbance of metabolic activation of 6-TG, e.g. by an enhanced rate of hypoxanthine phosphoribosyltransferase gene mutation in *Dnmt1*^*−/−*^ ES cells^31^. Furthermore, similar to the results of the 6-TG sensitivity assay, one randomly picked clone from each type of the stable ES clones all showed expression level-dependent rescue of MNNG resistance by *Dnmt1*^*−/−*^ ES cells through ectopic expression of Dnmt1 or its variants (Supplementary Fig. S1). Note that MMR deficient phenotypes of both the MNNG-resistance and the MSI of *Dnmt1*^*−/−*^ ES cells were attenuated in comparison with *Msh2*^30^ and *Msh6* deficiency^22^ ES cells, respectively (please see Discussion).

#### Analysis of MSI

The effects of ectopically-expressed Dnmt1 and variants on MMR functioning were also studied by MSI analysis. Genomic DNA was extracted from the stable ES clones cultured without feeder cells for 50 days and subjected to MSI analysis as previously described^21^. Notably, due to the fluctuating nature of mutation events, the MSI phenotype of a given cell population is referred to by the length alterations accumulated in some microsatellite markers, but not all of the markers analyzed. For comparison, three independent subclones of both the original WT and *Dnmt1*^*−/−*^ ES cells were also included in the analysis. As expected, the combined data from analysis of three independent clones of each type exhibited a trend in which the microsatellite markers were more stable in the high-level clones than in the low-level clones. As shown in Table 1, two of the microsatellite markers, D1Mit36 and D7Mit91, showed significantly higher frequencies of novel allele occurrence in the *Dnmt1*^*−/−*^ samples than the WT. Significantly, while all of the low-level clones still manifested the MSI phenotypes, the genomes of the high-level clones were more stable with respect to the microsatellite markers analyzed (Table 1). Thus, consistent with the test results of 6-TG sensitivity, MSI analysis of the different ES clones further demonstrated that Dnmt1 and variants exhibited expression level-dependent roles, but DNA methylation activity- and PCNA binding-independent roles, in MMR.

### Chromatin loading of Dnmt1/variants and proper MMR function

#### Chromatin loading of enzymatically active and inactive Dnmt1

We next examined the correlation between the MMR function of Dnmt1 and its chromatin loading. Previously, it was shown that in 15~30% of asynchronous mammalian cells, apparent Dnmt1 foci formed as a result of association of Dnmt1 with pericentric heterochromatin for the maintenance methylation of DNA during its replication at the late S phase^12^,^27^,^32^. In contrast, it has been shown previously that the inactive Dnmt1 variant *MG*^*C1229S*^ was mislocalized at pericentric heterochromatin when stably expressed in *Dnmt1*^*−/−*^ ES cells^32^. Mislocalization of the inactive Dnmt1 variant was determined to be independent of changes associated with the cell cycle and DNA synthesis, and thus was suggested to be a result of recruitment of the inactive Dnmt1 variant by the residual ^m^CpG prevalent in the pericentric heterochromatin in *Dnmt1*^*−/−*^ ES cells^32^. The disparities in 6-TG resistance, as well as MSI incidence between the stable ES clones expressing “high-levels” and “low-levels” of different types of Dnmt1 variants (Fig. 2A and Table 1), prompted us to determine whether the distribution patterns of the exogenous Dnmt1 and variants on the chromatin correlated with the efficiencies of MMR in these cells.

**Figure 3:**
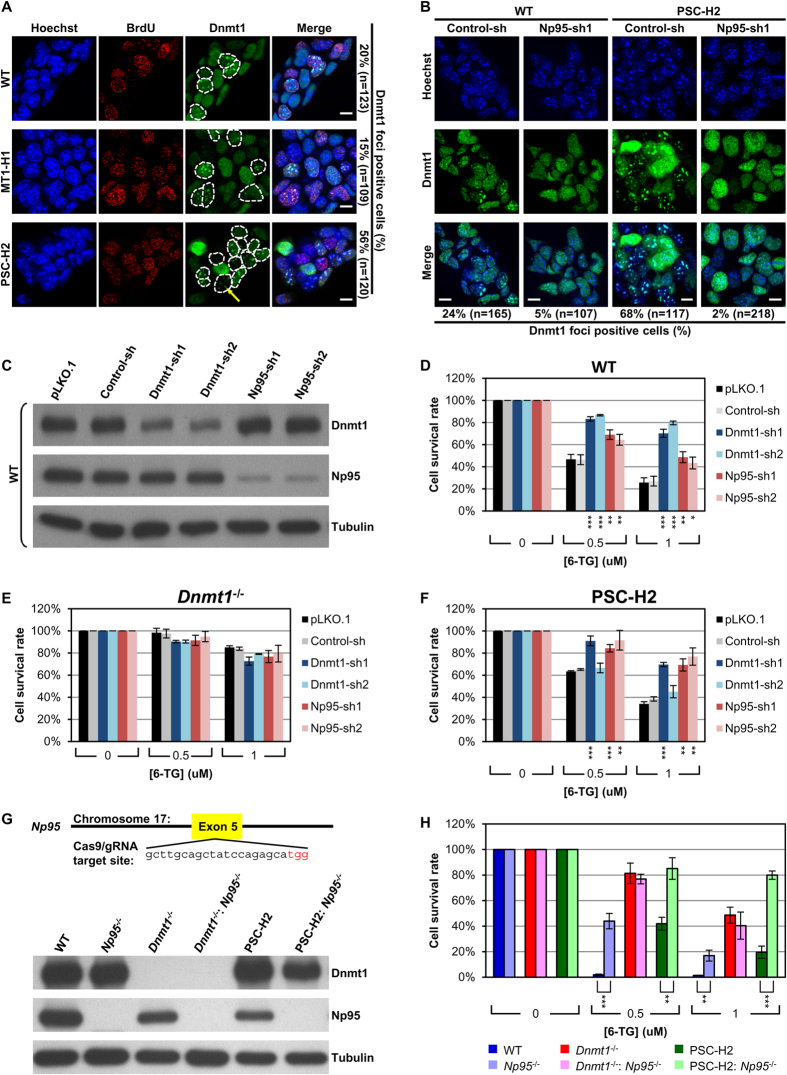
Np95-mediated chromatin accumulation of Dnmt1 and the PSC variant are required for the sensitivity of ES cells to 6-TG. (**A**) Enriched chromatin accumulation of the PSC variant. As shown, 15~20% of WT cells and MT1-H1 cells contain Dnmt1 foci (indicated by the dashed circles, green), coinciding with the dense chromatin (Hoechst staining, blue), which are also in S phase (BrdU labeled, red). For PSC-H2 cells, however, this percentage was increased to ~56% with some of the cells containing Dnmt1 foci not in S phase (the yellow arrow). (**B**) Requirement of Np95 for chromatin loading of Dnmt1. Similar analyses as in (**A**) were carried out for WT and PSC-H2 cells after transfection with either the Np95-sh1 or the Control-sh expressing plasmid. In both WT and PSC-H2 cells, the Dnmt1 foci disappeared after RNAi knockdown of Np95 by the Np95-sh1 shRNA. (**C**) Knockdown efficiencies of two shRNAs each targeting Dnmt1 or Np95. The WT ES cells were transfected with the vector pLKO.1 or plasmids expressing the Control-sh, Dnmt1-sh1, Dnmt1-sh2, Np95-sh1, and Np95-sh2 shRNA(s). The protein levels of Dnmt1 and Np95 were analyzed by Western blotting. (**D,E,F**) WT (**D**), *Dnmt1*^*−/−*^ (**E**), and PSC-H2 (**F**) cells were transfected with pLKO.1 vector or plasmids expressing the Control-sh, Dnmt1-sh1, Dnmt1-sh2, Np95-sh1 and Np95-sh2 shRNA(s), respectively. The cells were then subjected to 6-TG sensitivity assays. The data are exhibited as mean ± SEM (n = 3 in duplicate) compared to the Control-sh group. (**G,H**) Targeted deletion of *Np95* in the WT, *Dnmt1*^*−/−*^, and PSC-H2 cells. CRISPR/Cas9-mediated gene disruption was conducted to generate cell lines (*Np95*^*−/−*^, *Dnmt1*^*−/−*^: *Np95*^*−/−*^ and PSC-H2: *Np95*^*−/−*^) carrying homozygous deletion of Np95 in WT, *Dnmt1*^*−/−*^, and PSC-H2, respectively. The Cas9/gRNA target site and Western blotting patterns of Dnmt1 and Np95 of the genetically engineered cells are shown in (**G**). The cells were then subjected to 6-TG sensitivity assay and the data exhibited as mean ± SEM (n = 3 in duplicate) are shown in (**H**). Scale bar = 10 μm. Tubulin, the loading control. Student’s *t* test, *p < 0.05; **p < 0.01; ***p < 0.001.

As exemplified in Fig. 3A, the patterns of foci formation of the active ectopic Dnmt1 and its Q162E variant (Supplementary Fig. S2) in the stable clones were similar to the endogenous Dnmt1 in WT ES cells, regardless of their expression levels. The proportions of Dnmt1 foci-positive WT ES cells and MT1-H1 cells were 20% and 15%, respectively (Fig. 3A). In contrast, Dnmt1 foci appeared in much larger percentages of the stable ES cells ectopically expressing the inactive Dnmt1 PS or PSC variants (shown for PSC-H2 in Fig. 3A and the remainder in Supplementary Fig. S2), independently of their expression levels. The 56% of cells with foci formation by the inactive Dnmt1 variants might be attributed to mislocalization to the pericentric heterochromatin containing relatively high amounts of ^m^CpG, as previously suggested^32^. Alternatively, the inactive Dnmt1 variants could be trapped for lengthy periods at the hemi-methylated DNA during replication due to their deficiency in the maintenance DNA methylation^17^. In either case, the data of Fig. 3A indicates that overloading of inactive Dnmt1 on chromatin was not an obstacle for proper MMR functioning. In addition, defective MMR was rescued by strong expression of the Q162E variant of Dnmt1 (Fig. 2A and Table 1). Therefore, we reason that coupling of Dnmt1 with the replication machinery through PCNA-interaction is neither required for its loading on chromatin nor its function in MMR.

#### Induction of 6-TG resistance by knockout or RNAi knockdown of either Dnmt1 or Np95

The hemi-^m^CpG-binding protein Np95 was shown to be responsible for recruiting Dnmt1 during DNA replication for the maintenance of DNA methylation^14^,^15^. Further, without affecting the cellular content of Dnmt1, deficiency of Np95 caused DNA hypomethylation by reducing the chromatin loading of Dnmt1^14^,^15^. To test whether the overloading of the enzymatically-inactive Dnmt1 variants on chromatin were also mediated through the hemi-^m^CpG-bound Np95, the distributions of Dnmt1 and the PSC variant in WT and PSC-H cells were monitored by immunostaining after RNAi knockdown of Np95. As shown in Fig. 3B, both the Dnmt1 foci in WT cells and the foci of the overloaded-PSC variant in PSC-H2 cells disappeared upon knockdown of Np95. This suggests that like endogenous Dnmt1, the long-lasting and overloaded inactive PSC variant was also trapped at the hemi-methylated DNA bound with Np95.

To test the hypothesis that chromatin loading of Dnmt1 was required for its function in MMR, the levels of 6-TG sensitivity for WT ES cells were examined after RNAi knockdown of either Dnmt1 or Np95, the efficiencies of which were monitored by Western blotting (Fig. 3C). While the cellular amount of Dnmt1 was unaltered in the Np95 knockdown cells and *vice versa*^14^,^15^ (Fig. 3C), knockdown of either Dnmt1 or Np95 increased 6-TG resistance of the WT ES cells (Fig. 3D). In addition, RNAi knockdown of either Dnmt1 or Np95 had no further effects on the level of 6-TG resistance of the *Dnmt1*^*−/−*^ ES cells (Fig. 3E), suggesting that there were no off-target effects of the shRNAs on 6-TG resistance, as well as indicating that the requirement of Np95 for proper MMR functioning was epistatic to that of Dnmt1.

Dnmt1-sh2 targets to the 3′-UTR of the endogenous Dnmt1 mRNA, but the same target sequence is absent from the mRNAs of exogenous Dnmt1/variants. As a result, RNAi knockdown of Dnmt1 or Np95, other than Dnmt1-sh2, in PSC-H2 cells also attenuated the MMR function rescued by the high-level of the exogenous PSC variant (Fig. 3F), further supporting that Np95-mediated chromatin loading of Dnmt1 was required for proper MMR functioning.

The essential role of Np95 in Dnmt1-dependent MMR functioning was also addressed by CRISPR/Cas9-mediated gene disruption of *Np95* in WT, *Dnmt1*^*−/−*^, and PSC-H2 cells (Fig. 3G). As shown in Fig. 3H, *Np95*^*−/−*^ as well as PSC-H2: *Np95*^*−/−*^ cells showed a significant increase in 6-TG resistance when compared to the parental cells. In contrast, double knockout of *Dnmt1* and *Np95 (Dnmt1*^*−/−*^: *Np95*^*−/−*^) exhibited no further increase in 6-TG resistance in comparison to the parental *Dnmt1*^*−/−*^ cells (Fig. 3H). These results further strengthen our argument that Np95 and Dnmt1 act in an epistatic manner for proper *in vivo* MMR functioning.

### Dnmt1-dependent chromatin loading of the MMR proteins

#### Inefficient chromatin loading of MMR proteins in *Dnmt1* ^*−/−*^ ES cells

**Figure 4:**
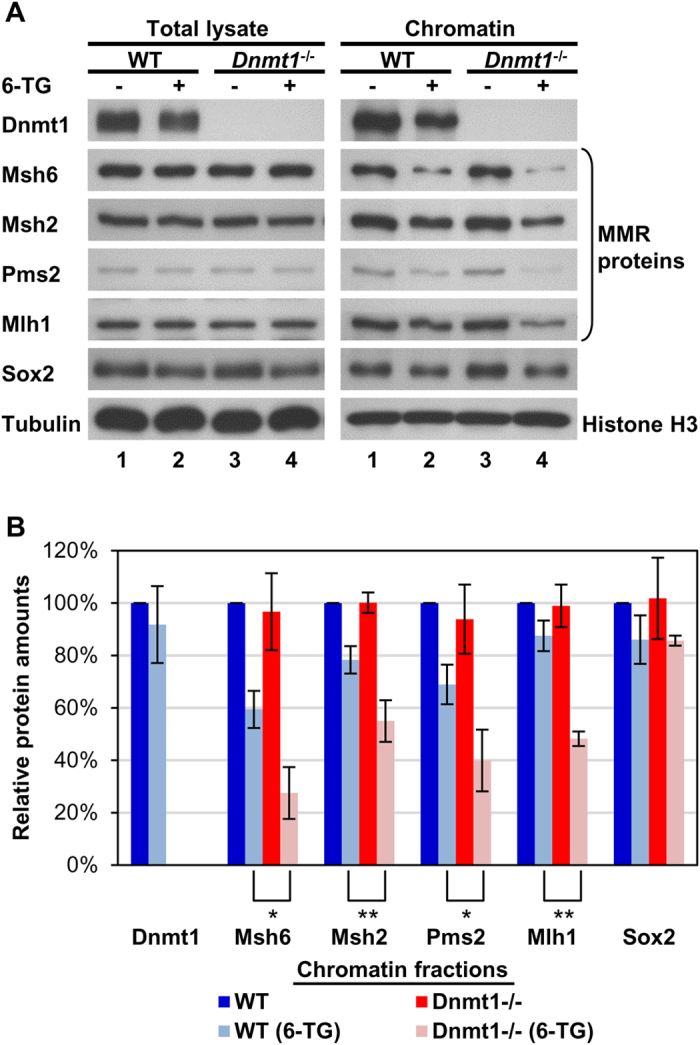
Inefficient chromatin loading of MMR proteins in *Dnmt1*^*−/−*^ ES cells. (**A**) Western blotting patterns of the levels of different MMR proteins in the total lysates and chromatin fractions of WT and *Dnmt1*^*−/−*^ ES cells with or without 6-TG treatment. Tubulin and histone H3 were used to normalize loading for the total lysates and the chromatin fractions, respectively. (**B**) Quantitative analysis of the relative amounts of the different MMR proteins in the chromatin fractions, mean ± SD (n = 3) as determined by Western blotting shown in (**A**) and Supplementary Fig. S3. Note the significantly reduced chromatin loading of the MMR proteins, but not the transcriptional factor Sox2, in the 6-TG treated *Dnmt1*^*−/−*^ cells in comparison to 6-TG treated WT cells. Student’s *t* test, *p < 0.05; **p < 0.01.

We next examined the accessibilities of the 6-TG-incorporated chromatin to the MMR proteins in WT and *Dnmt1*^*−/−*^ ES cells by Western blotting of the total cell lysates and the chromatin fractions^33^,^34^. As shown, the amounts of MMR proteins in the total lysates were not affected by depletion of Dnmt1 or by treatment with 6-TG (Fig. 4A, left panels). In the chromatin fractions, a basal chromatin association of MMR proteins was observed in WT and *Dnmt1*^*−/−*^ ES cells in the absence of 6-TG treatment (Fig. 4A, lanes 1 and 3 in the right panels). This result is consistent with previous studies using different chromatin preparation methods^33^,^34^, which could be the basis for a setup for an on-call system for mismatch signals^35^. Notably, similar levels of the basal chromatin association of MMR proteins between WT and *Dnmt1*^*−/−*^ ES cells (Fig. 4A, comparing lanes 1 and 3 in the right panels) indicated that it was independent of the presence of Dnmt1.

However, in contrast to the basal association of MMR proteins with chromatin, we observed Dnmt1-dependent chromatin loading of MMR proteins under treatment of 6-TG (comparing lanes 2 and 4 in the right panels of Fig. 4A); a condition in which the genome was full of DNA mismatches and known to activate MMR functioning within the chromatin environment^3^,^22^. Notably, 6-TG treatment of WT ES cells resulted in decreases of different proteins in the chromatin fractions (Fig. 4B and Supplementary Fig. S3, by 10~20% for Dnmt1, Msh2, Mlh1 and Sox2; and by 30~40% for Msh6 and Pms2). Some of these decreases could be in part due to the 6-TG-induced cell cycle arrest at G2 phase^36^ of the originally asynchronous ES cells, the majority of which were in S phase (Fig. 3A). For example, the level of histone H3, which we used as the gel loading control in Fig. 4A, might be higher in the chromatin samples from 6TG-treated cells (G2 phase) than those from the control ES cells (S phase), since DNA replication in eukaryotes required nucleosome disruption ahead of the replication fork^37^. Also, it was shown before by others that the *in vitro* mismatch binding activities of MMR proteins in nuclear extracts from G2 phase cells were significantly lower than those in nuclear extracts from S phase cells, which could be due to cell cycle-dependent post-translational regulation of the MMR proteins^38^. In any case, the further significant decreases of MMR proteins in the chromatin fractions of *Dnmt1*^*−/−*^ ES cells following 6-TG treatment (right panels of Fig. 4A, Supplementary Fig. S3 and histogram in Fig. 4B) demonstrates that MMR proteins are more efficiently loaded onto the 6-TG-damaged chromatin in the presence of Dnmt1.

#### Physical interaction between Dnmt1 and the MutSα complex

**Figure 5:**
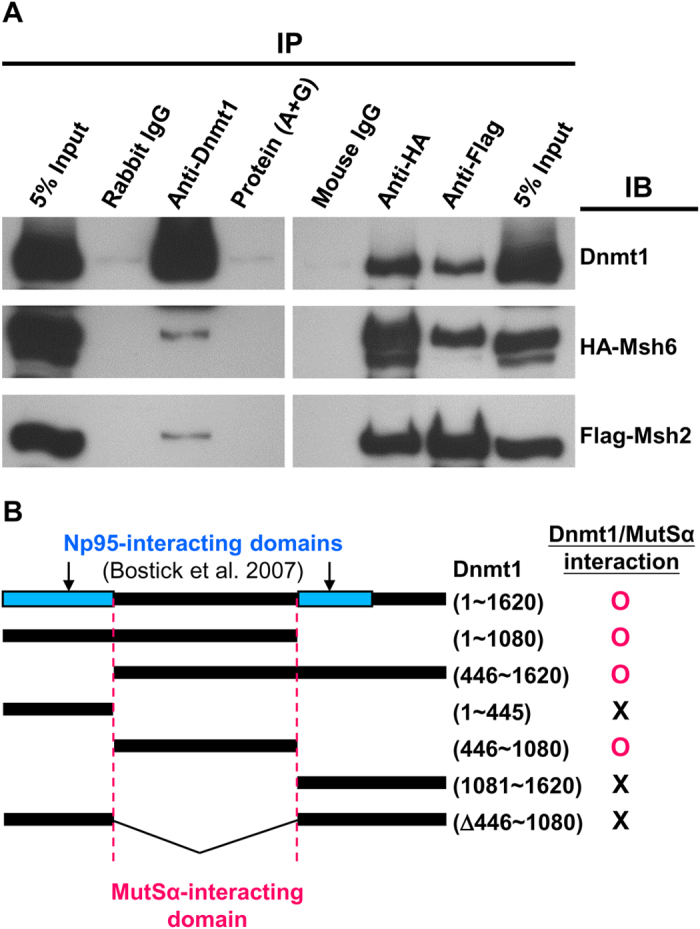
Physical interaction between Dnmt1 and the MutSα complex. (**A**) The potential for physical interaction between Dnmt1 and MutSα complex was examined by immunoprecipitation (IP) coupled with Western blotting (IB). Extracts from 293T cells co-expressing Dnmt1, HA-Msh6 and Flag-Msh2 were immunoprecipitated with control rabbit IgG, mouse IgG, anti-Dnmt1, anti-HA or anti-Flag, and then analyzed by IB using the latter three antibodies. The detection by IB of HA-Msh6 and Flag-Msh2 in the anti-Dnmt1 precipitate, as well as detection of Dnmt1 in the anti-HA (Msh6) or anti-Flag (Msh2) precipitate, indicates that Dnmt1 physically interacts with the MutSα complex. (**B**) Domain(s) of Dnmt1 interacting with the MutSα complex. The domain(s) was mapped by IP/IB assay of extracts from 293T cells co-expressing HA-Msh6, Flag-Msh2, and different fragments of Dnmt1. As summarized in the diagram, the IP/IB data (see Supplementary Fig. S4) show that Dnmt1 interacts with the MutSα complex through its central region (a.a. 446~1080), which is non-overlapping with the Np95-interacting domains of Dnmt1.

In view of these observations, we examined whether Dnmt1 physically interacts with MMR proteins. Since the MutSα complex accounts for the major activity of mismatch surveillance^7^, we overexpressed Dnmt1 in 293T cells with tagged MutSα components, HA-Msh6 and Flag-Msh2. Immunoprecipitation (IP) assays of the total extracts from the transfected cells were then carried out with the use of anti-Dnmt1, anti-HA or anti-Flag antibodies (Fig. 5A). Indeed, Dnmt1 interacted with the heterodimeric MutSα complex (Fig. 5A). Interestingly, Dnmt1 can be recruited to the DNA repair sites through its interactions with the DNA damage response machinery, independently of the cell cycle phase^39^,^40^. In addition, Ding *et al.*^41^ recently reported that H_2_O_2_ treatment of cells could induce interactions of the epigenetic proteins DNMT1, SIRT1 and EZH1 with the DNA repair proteins MSH2, MSH6 and PCNA in an S-phase independent fashion and that it also potentiated the interaction between DNMT1 and the MutSα complex.

This physical interaction was further validated by mapping of the MutSα-interacting domain(s) of Dnmt1. As shown in Fig. 5B and Supplementary Fig. S4, IP assays of 293T cell extracts containing different Dnmt1 fragments and HA-Msh6/Flag-Msh2 revealed that the central part of Dnmt1 (a.a. 446~1080) was required and sufficient for Dnmt1 interaction with the MutSα complex (Fig. 5B). Interestingly, this domain is non-overlapping with the two Np95-interacting domains of human DNMT1 previously mapped by Bostick *et al.*^14^.

### The impact of the level of genomic 5-mC on MMR efficiency

**Figure 6:**
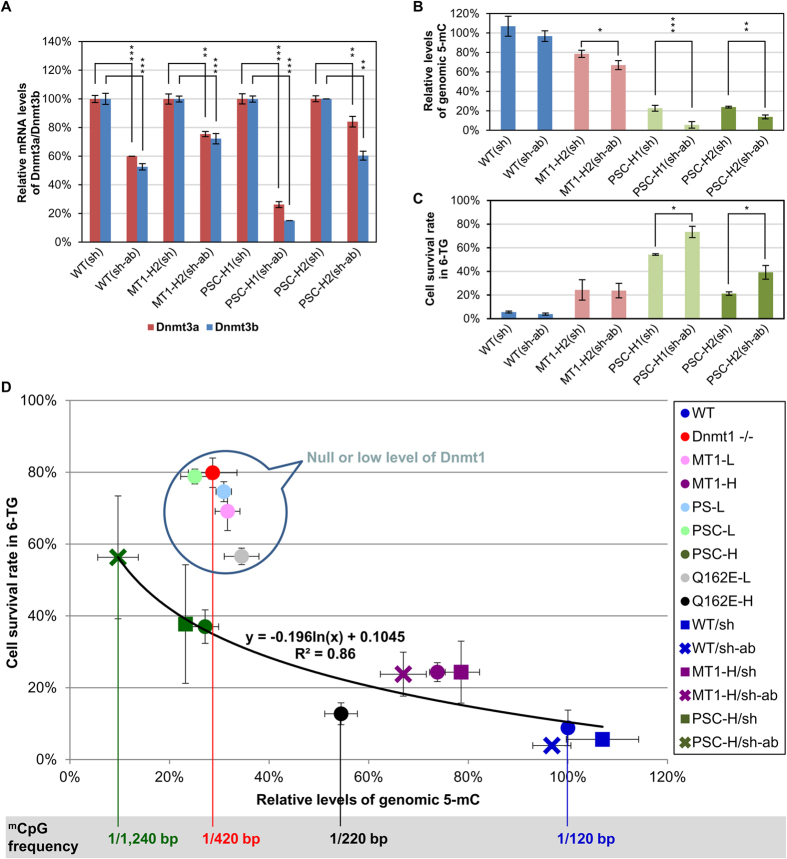
Impact of genomic 5-mC on MMR in ES cells. (**A**) Four cell lines WT, MT1-H2, PSC-H1 and PSC-H2, were used to generate stable cell lines expressing either a control shRNA (sh) or shRNAs (sh-ab) capable of double-knockdown of Dnmt3a/Dnmt3b. The levels of Dnmt3a and Dnmt3b in the different cell lines were evaluated by quantitative RT-PCR and are shown as mean ± SEM (n = 3 in duplicate). (**B**) The relative levels of genomic 5-mC in these cell lines were analyzed by HPLC and the results of mean ± SD (n = 3) are shown in percentages relative to the WT cells. (**C**) MMR functioning in these cell lines was evaluated by the 6-TG sensitivity assay and the results are shown as mean ± SEM (n = 3 in duplicate). Student’s *t* test for (**A**) and (**B**) and paired Student’s *t* test for (**C**), *p < 0.05; **p < 0.01; ***p < 0.001. (**D**) The relationship between the efficiency of Dnmt1-dependent MMR and the genomic content of 5-mC. Survival rates in 0.5 μM 6-TG of the different types of ES cells are the mean ± SEM adapted from Figs 2A and 6C, and the genomic contents of 5-mC are the mean ± SD adapted from Figs 1D and 6B. A regression curve of the survival rates as a function of the 5-mC contents is plotted for the WT cells and the different high-level ES clones (R^2^ = 0.86). An accelerated increase of cell survival under 6-TG treatment appears when the ^m^CpG frequency is lower than 1/220 bp.

Since the local chromatin accumulation of Np95 *in vivo*, and consequently that of Dnmt1, was dependent on the presence of hemi-methylated DNA^14^,^15^ (Fig. 3), we evaluated the dependence of MMR on the levels of genomic 5-mC in WT and different stable ES clones. For this, WT, MT1-H2, PSC-H1 and PSC-H2, all of which exhibited proper MMR functioning and high 6-TG sensitivity, were used to generate cell lines stably expressing either control shRNA (sh) or shRNAs (sh-ab) capable of double-knockdown of Dnmt3a/Dnmt3b. The knockdown efficiencies of Dnmt3a/Dnmt3b in these stable cell lines were determined by quantitative RT-PCR to be in the range of 20%~90% (Fig. 6A). The genomic 5-mC contents of these clones were determined by HPLC. As shown in Fig. 6B, the originally low 5-mC contents of the two PSC-H clones (~30% of WT) were further reduced to 6% (PSC-H1) and 14% (PSC-H2) upon double-knockdown of Dnmt3a/Dnmt3b. In contrast, the high levels of 5-mC in WT (~100%) and MT1-H2 (~77% of WT) cells were only moderately affected by double-knockdown of Dnmt3a/Dnmt3b (Fig. 6B), due to the relatively high Dnmt1 activities in these cells.

The MMR activities in these cell lines were evaluated by 6-TG sensitivity. Significant loss of MMR efficiency, as reflected in increased 6-TG resistance, was observed in the two PSC-H(sh-ab) cell lines in comparison to the corresponding PSC-H(sh) cells (Fig. 6C). In contrast, double-knockdown of Dnmt3a/Dnmt3b in the WT and MT1-H2 cells had no effects on their 6-TG sensitivities. Together, the data of Fig. 6 suggest that reduction of Dnmt3a/Dnmt3b independently had little effect on MMR, whereas it seems a decrease in genomic 5-mC content to below a certain threshold, such as 14% as in PSC-H1(sh-ab) and PSC-H2(sh-ab) (Fig. 6B), affects MMR efficiency. Most likely, this was due to the requirement for a minimum number of hemi-^m^CpG docking sites for the Np95-Dnmt1-MMR complex on the chromatin.

The impact of the genomic content of 5-mC, which determines the density of hemi-^m^CpG on the replicating DNA, on the efficiency of post-replicative MMR surveillance was further deduced from our data of different ES cell lines carrying different levels of Dnmt1 or its variants (Fig. 6D). In general, CpG content accounts for 1% of dinucleotides in the mouse genome^42^ and nearly 85% of the CpG sites are methylated^43^. This average frequency of ^m^CpG (~1 in 120 bp) ensures efficient MMR surveillance of the newly-synthesized DNA through the axis of the hemi-^m^CpG-Np95-Dnmt1-MMR complex. As the genomic content of 5-mC is reduced to ~30% of the wild-type level in the *Dnmt1*^*−/−*^ ES cells (Fig. 1D), the average frequency of the ^m^CpG sites becomes ~1 in 420 bp and MMR is impaired (Fig. 2 and Table 1). Proper MMR of this genome could be partially restored by a sufficiently high level of the inactive Dnmt1. For example, in the PSC-H and PSC-H(sh) cell lines, it is restored to ~60% [(Δ^*Dnmt1−/−*_PSC-H^/Δ^*Dnmt1−/−*_WT^) = (80–37%)/(80–9%)] of the WT ES cells (Fig. 6D). Although the MT1-H and PSC-H clones ectopically expressed nearly identical amounts of Dnmt1 and its inactive variant, respectively (Fig. 1A), the significantly lower genomic content of ^m^CpG in PSC-H clones led to an increase in 6-TG resistance in comparison to the MT1-H clones (Fig. 6D). A further decrease in the average frequency of ^m^CpG on the genome to ~1 in 1,240 bp, as in the PSC-H(sh-ab) cell lines (Fig. 6D), significantly decreased the efficiency of MMR surveillance to ~30% [(Δ^*Dnmt1−/−*_PSC-H(sh-ab)^/Δ^*Dnmt1−/−*_WT^) = (80–56%)/(80–9%)] of the WT ES cells (Fig. 6D). Thus, the effectiveness of post-replicative DNA surveillance on the mouse genome by MMR indeed depends on a sufficiently high level of Dnmt1, as well as the frequency of ^m^CpG.

## Discussion

DNA hypomethylation of mammalian genomes is associated with elevated mutation rates, chromosomal instability and tumor susceptibility, as shown by analysis of cells as well as mice expressing reduced levels of Dnmt1^28^,^31^,^44^. Furthermore, Dnmt1 appears to participate in MMR, although the mechanisms are unclear^21^,^22^,^23^,^24^. Despite these studies, the causative roles of DNA methylation and Dnmt1 have remained to be elucidated. In the present study, we demonstrate that mammalian post-replicative DNA MMR cooperates with a hemi-^m^CpG-Np95-Dnmt1 axis in living cells to enhance the efficiency of MMR surveillance of the mammalian genome at the epigenetic level through the cellular amounts of Dnmt1, as well as by the frequency of ^m^CpG on the mouse genome. Consequently, the genome instability and tumor susceptibility associated with reduced Dnmt1 and DNA methylation are caused in part by impairment of MMR functioning. This scenario is consistent with the phenotypes of MMR-deficient mice, which include genome instability and predisposition to cancer^45^, as well as global DNA hypomethylation in tumor cells^46^. Clinically, the effect of DNA methylation status on MMR efficiency might need to be taken into consideration in cancer therapies using chemotherapeutic drugs that are MMR-dependent.

Notably, MMR impairment in Dnmt1-deficient cells has been hypothesized to be the result of imbalanced gene expression due to genome-wide hypomethylation in these cells. It has also been argued that MSI and 6-TG tolerance could also result from DNA hypomethylation-associated chromosome instability and disturbances involved in the metabolic activation of 6-TG, respectively^24^,^31^,^44^. Moreover, an indirect effect of decreased abundance of MMR components was observed in human somatic and tumor cells with Dnmt1 deficiencies, in which a DNA damage response was activated by the loss of Dnmt1, ultimately leading to cell death^17^,^18^. Here, we show that in ES cells, which can proliferate normally with complete inactivation of Dnmt1, proper functioning of post-replicative MMR requires Dnmt1, independently of its catalytic activity as well as its interaction with PCNA. More importantly, the phenotypes of MSI, 6-TG tolerance, as well as MNNG resistance of *Dnmt1*^*−/−*^ ES cells, could be rescued by high-levels of the inactive Dnmt1 variant PSC (Supplementary Fig. S1), underscoring an essential role for Dnmt1 acting directly in vertebrate MMR *in vivo*. This synergy of Dnmt1 and MMR, as directed by the hemi-^m^CpG sites bound with Np95, endows vertebrate CpG methylation with a new biological significance, i.e. priming DNA replication for MMR quality control.

It should be noted here that PCNA is also essential for MMR initiation through its interaction with the MMR proteins^47^,^48^,^49^. While an initial observation of interaction of Dnmt1 with PCNA^13^ suggested a possible link between Dnmt1 and the replication complex for the maintenance DNA methylation by Dnmt1, later studies have shown that the Dnmt1-PCNA interaction is highly dynamic and not strictly required for maintaining global genome methylation^27^,^50^. Our data presented in Fig. 2A, Table 1 and Supplementary Fig. S1 also indicate that Dnmt1 with a mutation in the PCNA-interacting domain could still function in MMR. Thus, we suggest here that the MMR complex(es) recruited to the hemi-^m^CpG sites on chromatin by the Np95-Dnmt1 complex act in parallel with the PCNA-MMR complex^51^,^52^,^53^, providing a double level of insurance in surveillance of DNA mismatch(es). This is in agreement with the lower MNNG resistance and MSI incidence observed in *Dnmt1*^*−/−*^ ES cells compared to mouse cells/tissues with defective MMR genes^22^,^30^,^45^ (Fig. 2B).

**Figure 7:**
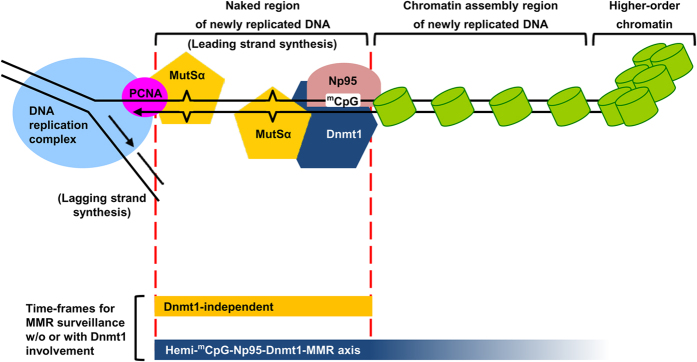
A Model for the dependence of MMR on the hemi-^m^CpG-Np95-Dnmt1 axis. During DNA replication, chromatin assembly on the newly-synthesized DNA helix takes place ~250 bp behind the replication fork, and it could block the mismatch recognition of MMR in the absence of Dnmt1 (the yellow bar at bottom). Cooperation between maintenance DNA methylation and MMR through the hemi-^m^CpG-Np95-Dnmt1-MMR axis can proceed in parallel to the action of the PCNA-MMR complex to detect DNA mismatches on the post-replicative DNA. Differing from Dnmt1-independent MMR, e.g. that by the PCNA-MMR complex, hemi-^m^CpG-Np95-Dnmt1 axis-directed MMR can significantly extend the time-frame of MMR surveillance from the duration of post-replicative DNA as a naked helix until after nucleosome assembly (the blue bar at bottom). For more details, see text.

As depicted in our cartoon model of MMR surveillance of vertebrate genomes (Fig. 7), the post-replicative hemi-^m^CpG site(s) bound with Np95 provide a new item on the checklist for enhancing the efficiency of genome surveillance. Notably, MMR and chromatin assembly are mutually exclusive^54^,^55^,^56^. Moreover, nucleosomes are assembled ~250 bp behind the DNA replication fork^56^ (Fig. 7, top cartoon model), and this would limit the time-frame available for MMR surveillance after DNA replication (Fig. 7, yellow bar). In contrast, the action of the Np95-Dnmt1-mediated DNA methylation system taking place in S phase and G2 phases allows enough time for the maintenance of densely methylated heterochromatin^14^,^15^,^57^, which also appears to extensively crosstalk with the histone code. In particular, Np95 not only binds hemi-^m^CpG but also interacts with the histone modifications^58^,^59^, and maintenance of vertebrate genome-wide DNA methylation requires SNF2-like proteins with chromatin-remodeling activities^60^. Therefore, recruitment of MMR functioning to the hemi-^m^CpG-Np95 complex on the newly-replicated DNA regions by Dnmt1, along with Dnmt1’s function of maintenance DNA methylation, would enhance replication fidelity. This is achieved not only by concentrating MMR surveillance on the newly replicated DNA, but also by expanding the time-frame for MMR surveillance aided through the crosstalks between the Np95-Dnmt1 complex, the histone codes, and the chromatin remodeling process (Fig. 7, blue bar).

## Methods

### Cell culture, DNA transfection, and generation of stable overexpression clones/knockdown cell lines

Wild-type mouse ES cell line J1 and its descended *Dnmt1*^*−/−*^ clone with homologous *Dnmt1*^*c*^ alleles were kindly provided by Dr. En Li (Novartis). The ES cells were cultured as described previously^16^ with some modifications. The cells were grown on gelatin-coated plates and in medium with leukemia inhibitory factor (ESGRO, Millipore). The 293T cell line was obtained from ATCC and cultured in Dulbecco’s Modified Eagle’s Medium plus 10% fetal bovine serum. Plasmid DNA transfection was performed with the use of Lipofectamine 2000 (Invitrogen). To generate stable clones, the individual plasmid constructs were transfected and the transfected cells were selected by blasticidin (InvivoGen). The expression levels of Dnmt1 or its variants in the stable clones were evaluated by Western blotting of the total cell extracts with tubulin as the loading control. For analysis of low-level clones, only one-fifth of the WT extract was loaded in the gel as the control (see Fig. 1A).

To generate stable cell lines with double-knockdown of Dnmt3a and Dnmt3b, different shRNA-expressing plasmid constructs targeting Dnmt3a or Dnmt3b (three for each) were pooled together and co-transfected. The transfected cells were selected by puromycin (InvivoGen) to generate stable clones and 5–6 puromycin-selected clones were pooled to establish the individual stable cell lines.

### Expression plasmid constructs of *Dnmt1* minigene and its variants

The wild-type *Dnmt1* minigene (MT1) was amplified from the cDNA pool prepared from the J1 ES cells and cloned into the expression vector resulting in pCI-MT1-IRES-Bsd (un-published, a derivative from pCI of Promega). The CMV promoter drives a bicistronic transcript containing MT1 and an IRES-connected blasticidin-resistance gene. The plasmid constructs expressing different Dnmt1 variants were generated using the QuikChange Site-Directed Mutagenesis Kit (Stratagene). The Q162E variant contained a substitution of glutamic acid for glutamine^27^. In the PS variant, the enzymatic activity of Dnmt1 was abolished by replacing Cys1229 at the catalytic center with serine^26^. The PSC variant contained a serine insertion before Cys1229 to mimic the inactive DNA methyltransferase homologue in fission yeast^25^.

### Expression plasmid constructs of shRNAs

The effective shRNAs targeting Np95, Dnmt1, Dnmt3a or Dnmt3b were individually evaluated and selected from the lentiviral-based shRNA-expressing plasmid DNA library generated by the RNAi Consortium and prepared by the RNAi Core at Academia Sinica according to the results of quantitative RT-PCR (Dnmt3a or Dnmt3b) and Western blotting (Dnmt1 or Np95) analyses following transient transfection of the plasmid DNAs in ES cells for 2 days. The empty vector pLKO.1 and pLKO.1-sh plasmid expressing a shRNA (Control-sh) without targets in the mouse genome were used as controls. The RNAi Consortium Numbers (TRCNs) of the selected shRNA plasmid DNAs are the following: TRCN0000039482 and TRCN0000302343 for Np95-sh1 and Np95-sh2; TRCN0000039027 and TRCN0000225700 for Dnmt1-sh1 and Dnmt1-sh2; TRCN0000039034, TRCN0000039036 and TRCN0000039037 for Dnmt3a; TRCN0000071068, TRCN0000071070 and TRCN0000071072 for Dnmt3b.

For the purposes of proper processing of the shRNAs and generation of stable knockdown cell lines, DNA oligos encoding a control shRNA targeting the *lacZ* sequence (sh) and the six DNA regions encoding shRNAs targeting Dnmt3a or Dnmt3b with a high score of knockdown efficiency (<60% remaining) were chosen and individually re-cloned into the pCI.1-miR-EGFP-Puro expression vector (un-published, a derivative from pCI of Promega). The coding sequence of each of the seven shRNAs was embedded between the 5′ and 3′ flanking sequences of the murine miR-155 precursor^61^ within the artificial intron of the pCI.1-miR-EGFP-Puro expression vector. The CMV promoter in the plasmid directed co-expression of the shRNA(s) with a bicistronic transcript containing EGFP and an IRES-connected puromycin-resistance gene. Puromycin resistance was used for the selection of different stable ES clones and the intensity of EGFP fluorescence was used as an indicator of the expression level of the intronic shRNA(s).

### CRISPR/Cas9-mediated genome engineering

To knockout *Np95* by CRISPR/Cas9-mediated gene disruption, a guide RNA (gRNA) sequence in the 5′-G(N)_19_NGG-3′ framework targeting to the sequence within exon 5 of *Np95* (see Fig. 3G) was determined by using the online tool, http://crispr.mit.edu. The Np95-gRNA expression plasmid was constructed by ligation of the DNA oligos encoding the Np95-gRNA into the BsmBI double-cutting sites of pU6-sgRNA.pPuro vector (provided by the RNAi Core at Academia Sinica). ES cells were transfected with eSpCas9(1.1)^62^ (a gift from Feng Zhang, Addgene plasmid # 71814) and the Np95-gRNA expression plasmid. After selection by puromycin (2 ug/ml for 2 days) and recovery for 1 week, individual clones were picked and then screened by PCR-sequencing for the clones with homozygous frame-shift mutations. The targeted deletion of Np95 in the selected clones was also confirmed by Western blotting. Sequences of DNA oligos for Np95-gRNA expression plasmid construction and PCR-sequencing are given in Supplementary Table S1.

### Quantitative RT-PCR analysis

RNAs from different ES cell lines were extracted by TRIzol reagent (Invitrogen) followed by cDNA synthesis using the Superscript II Reverse Transcriptase (Invitrogen). The real-time PCR was performed on a LightCycler Nano instrument (Roche) with use of the Roche SYBR Green I Master Mix. All procedures followed the manufacturer’s instructions and the results are presented as mean ± SEM. Primer sequences are available in Supplementary Table S1.

### Genomic DNA preparation, bisulfite sequencing, and quantification of genomic 5-mC contents

For genomic DNA preparation, cells were collected by scraping from 10 cm culture dishes in 1 ml of lysis buffer (20 mM Tris-HCl, pH 8.0, 50 mM NaCl, 10 mM EDTA, 1% SDS, and 0.5 mg/ml proteinase K) and incubated at 55 °C overnight. The DNA was purified from the lysates by phenol extraction, ethanol precipitation and suspension in H_2_O. Bisulfite treatment of the genomic DNA was performed according to the manufacturer’s instructions for the EZ DNA Methylation-Gold Kit (ZYMO RESEARCH). PCR primers against the bisulfite-treated DNA for the amplification of the mouse skeletal *α–actin* and imprinted *H19* (region A) promoters were the same as described^63^,^64^. PCR was performed using EpiTaq HS DNA polymerase (TaKaRa), and the PCR products were cloned into pGEM-T Vector (Promega) for sequence analysis. In Fig. 1B,C, each line represents a single DNA methylation profiling result of bisulfite sequencing and twelve profiles of each genomic DNA sample were used to calculate the percentage of 5-mC in the CpG sites, as shown in the parentheses. The procedures for RP-HPLC (Reverse-phase high performance liquid chromatography) followed those described by Ramsahoye^65^. The DNA hydrolysates were analyzed using the Waters HPLC system with LiChrospher 100 RP-18 columns (Merck). Elution was performed with 50 mM (NH_4_)_2_PO_4_, pH 4.1 at room temperature at a flow rate of 1 ml/min. Each mean ± SD was derived from three independent DNA preparations and measurements.

### 6-TG or MNNG sensitivity assay

The 6-TG sensitivity assays were performed on a 24-well cell culture plate (TPP). Approximately 200 ES cells were seeded with 1 ml normal culture medium in each well and supplemented with different amounts of 6-TG (Sigma) as indicated. Except for transiently knocked-down cells on day 4, the cell viability was monitored on day 6 of 6-TG treatment by means of an alamarBlue assay (Invitrogen) according to the manufacturer’s instructions. The cell survival rates of the 6-TG treated wells were calculated as the fluorescence ratios of the 6-TG treated well over the control well without 6-TG treatment. The cell survival rates derived from three sets of duplicated experiments are presented in the histobar diagram as mean ± SEM. MNNG sensitivity assays were performed similarly to the 6-TG sensitivity assay, but approximately 1,000 ES cells were seeded and the cell viability was monitored on day 4 after MNNG (Chem Service) exposure according to the described procedure^30^. The curves of cell survival rates derived from four sets of duplicated experiments with increasing amounts of MNNG ranging from 0.05 μM to 0.8 μM are presented as mean ± SEM in Fig. 2B, while those derived from two sets of duplicated experiments treated with two doses of MNNG are presented in the histobar diagram as mean ± SEM in Supplementary Fig. S1.

### MSI analysis

The single-allele dilution PCR method was conducted for MSI analyses as described^21^,^66^,^67^,^68^. The five microsatellite markers analyzed in this study included three dinucleotide microsatellites (D1Mit36, D7Mit91 and D14Mit15) and two mononucleotide repeats (JH101 and JH102). The primer sequences are listed in the Supplementary Table S1. After PCR, microsatellite marker genotyping was carried out by the Sequencing Core Facility of the National Yang-Ming University Genome Research Center (YMGC).

### Antibodies

The antibodies used for immunostaining, immunoprecipitation, and Western blotting analyses included the following: α-Tubulin mouse monoclonal antibody (T5168, Sigma), BrdU rat monoclonal antibody (ab6326, Abcam), Dnmt1 rabbit polyclonal antibodies for immunostaining and immunoprecipitation (BAM-70-201, Cosmo Bio) and only for immunoprecipitation (ab87656, Abcam), Flag mouse monoclonal antibody (F3165, Sigma), HA mouse monoclonal antibody (clone 12CA5, Roche), Histone H3 rabbit polyclonal antibody (ab1791, Abcam), Msh2 mouse monoclonal antibody (NA27, Calbiochem), Msh6 mouse monoclonal antibody (610919, BD Biosciences), Mlh1 mouse monoclonal antibody (554073, BD Biosciences), Myc mouse monoclonal antibody (05–9724, Millipore), Pms2 mouse monoclonal antibody (556415, BD Biosciences), Sox2 goat polyclonal antibody (sc-17320, Santa Cruz), and UHRF1 (Np95) mouse monoclonal antibody (sc-373750, Santa Cruz).

### Immunostaining

Immunostaining with use of primary antibodies against different proteins (see above) and Alexa Fluor-conjugated secondary antibodies (Invitrogen) of the ES cells was carried out following standard procedures. Hoechst dye (B2261, Sigma) was used to stain the nuclear DNA after immunostaining. For 5-bromo-2′-deoxyuridine (BrdU, Roche) labeling of replicating cells, cells in culture were pulsed with 10 μM BrdU for 10 min before fixation and staining by anti-BrdU. For immunostaining of cells with transient knockdown of Np95 or Dnmt1, cells were transfected with the shRNA-expressing plasmid constructs on day 1 after seeding for 48 hr and then processed on day 3 for staining.

### Western blotting analysis of total cell lysates and chromatin fractions

ES cells with or without 1 μM 6-TG treatment for 24 hr were harvested and total cell lysates were collected by scraping the cells from the 10 cm culture dish in 500 ul RIPA buffer with protease inhibitor cocktail (Roche). To isolate the chromatin fractions, cells were permeabilized and washed in a digitonin (Wako)-containing buffer with protease inhibitor cocktail (Roche) and then treated with the crosslinking reagent DTSSP (Pierce) on the 10-cm culture dishes as described^33^. The total cell lysates and the chromatin fractions were then boiled in 2× Laemmli sample buffer and analyzed by SDS-PAGE and Western blotting with use of appropriate antibodies. An AlphaImager 2200 (Alpha Innotech) was used for quantitative analysis of the Western blotting results.

### Immunoprecipitation

For immunoprecipitation experiments, minigenes of mouse Msh2 and Msh6 were amplified from the cDNA pool from J1 ES cells and cloned into the pCI expression vector (Promega) with Flag or HA tag sequence in-frame at the 5′ end of the minigenes, resulting in pCI-Flag-Msh2 and pCI-HA-Msh6, respectively. The plasmids expressing the Dnmt1 deletion mutants or a series of N-terminal Myc-tagged Dnmt1 fragments were constructed by PCR-cloning using pCI-MT1-IRES-Bsd as the template and subcloning of the appropriate DNA fragments into the pCI expression vector (Promega). For the co-immunoprecipitation assay, 2 μg each of pCI-Flag-Msh2 and pCI-HA-Msh6 were co-transfected into 293T cells together with the individual plasmids expressing Dnmt1 or its fragments, as described above. On day 2 of transfection, the transfected cells were harvested in NP-40 buffer (10 mM HEPES-NaOH pH7.4, 142.5 mM KCl, 0.2% NP-40) containing the protease inhibitor cocktail (Roche). The supernatants obtained by centrifugation of the cell extracts at 12,000 rpm for 20 min were incubated with the appropriate antibodies or control IgGs at 4 °C for 2 hr. Protein A/G gel slurry (Pierce) pre-treated with 1% BSA in the NP-40 buffer was then added, and the samples were again incubated for 2 hr at 4 °C. After extensive washing of the beads with NP-40 buffer, the proteins bound to the beads were eluted by boiling in 2× Laemmli sample buffer and then subjected to SDS-PAGE and Western blotting analysis.

## Additional Information

**How to cite this article**: Wang, K.-Y. *et al.* Epigenetic Enhancement of the Post-replicative DNA Mismatch Repair of Mammalian Genomes by a Hemi-^m^CpG-Np95-Dnmt1 Axis. *Sci. Rep.* **6**, 37490; doi: 10.1038/srep37490 (2016).

**Publisher’s note:** Springer Nature remains neutral with regard to jurisdictional claims in published maps and institutional affiliations.

## Supplementary Material

Supplementary Information

## Figures and Tables

**Table 1:** Rescue of the MSI of *Dnmt1* ^*−/−*^ ES cells by high levels of Dnmt1/variants.

ES Clones	Markers				
	JH101	JH102	D1Mit36	D7Mit91	D14Mit15
WT	a0.5% (202)^b^	4.8% (227)	2.0% (205)	2.5% (239)	13.0% (207)
*Dnmt1*^*−/−*^	2.9% (204)	3.6% (252)	**9.3% (182)	*8.2% (220)	18.9% (190)
MT1-L	**5.4% (184)	*11.3% (204)	4.3% (185)	*6.9% (204)	*22.9% (153)
MT1-H	2.1% (195)	3.8% (186)	3.2% (221)	5.3% (245)	9.1% (208)
PS-L	2.0% (250)	3.8% (320)	*6.6% (228)	*7.1% (225)	16.1% (199)
PSC-L	***7.8% (205)	**13.1% (214)	2.7% (264)	2.8% (211)	13.7% (219)
PSC-H	0.0% (202)	3.4% (207)	1.5% (200)	3.0% (202)	14.7% (218)
Q162E-L	*3.4% (235)	3.8% (265)	4.5% (222)	2.6% (234)	12.9% (209)
Q162E-H	2.2% (89)	2.2% (93)	1.3% (79)	7.0% (86)	7.2% (69)

The microsatellite instabilities of the different stable ES clones are compared to the WT with the *p*-values calculated by Fisher’s exact test. *p < 0.05; **p < 0.01; ***p < 0.001.

^a^The percentage means the fraction of PCR reactions with novel alleles.

^b^The numbers in the parentheses represent successfully amplified PCR from three clones of each cell type, except that only one ES clone with high-levels of the exogenous Q162E variant (Q162E-H1) was available for analysis.
