# Supplementary material for: Epigenetic Enhancement of the Post-replicative DNA Mismatch Repair of Mammalian Genomes by a Hemi-mCpG-Np95-Dnmt1 Axis
Source: Sci Rep. 2016 Nov 25;6:37490. doi: 10.1038/srep37490 (PMC5122852; doi:10.1038/srep37490)
Supplement: Supplementary Information [file srep37490-s1.pdf]

Epigenetic Enhancement of the Post-replicative DNA Mismatch Repair of Mammalian Genomes by a Hemi-^{m}CpG-Np95-Dnmt1 Axis

Keh-Yang Wang, Chun-Chang Chen, Shih-Feng Tsai & Che-Kun James Shen

Inventory of Supplementary Information

Supplementary Figure S1. Expression level-dependent rescue of MNNG resistance of *Dnmt1*$^{-/-}$ ES cells by ectopic expression of Dnmt1 or its variants

Supplementary Figure S2. Enriched chromatin accumulation of the inactive Dnmt1 variants (PS and PSC)

Supplementary Figure S3. The replicated sets of Western blot images for the quantitative analysis of Fig. 4B

Supplementary Figure S4. Mapping of the MutSα-interacting domains of Dnmt1

Supplementary Table S1. DNA oligo sequences

Supplementary References

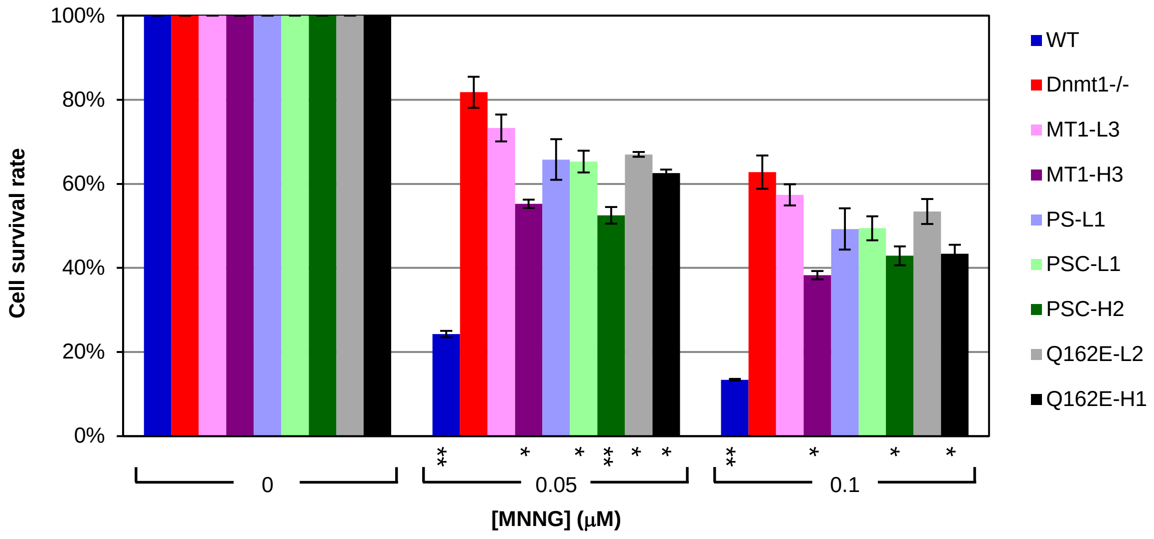

Supplementary Figure S1.

<u>Expression level-dependent rescue of MNNG resistance of *Dnmt1*$^{-/-}$ ES cells by ectopic expression of Dnmt1 or its variants</u>

One clone randomly picked from each type of the stable ES clones were treated with two doses of MNNG in the presence of O^6-benzylguanine. The cell survival rates derived from two sets of duplicated experiments are presented in the histobar diagram as mean ± SEM. The significances of the data were calculated by comparing the survival rates to that of the *Dnmt1*$^{-/-}$ ES cells (red bars). Student's *t* test, * $p < 0.05$; ** $p < 0.01$.

Supplementary Figure S2.

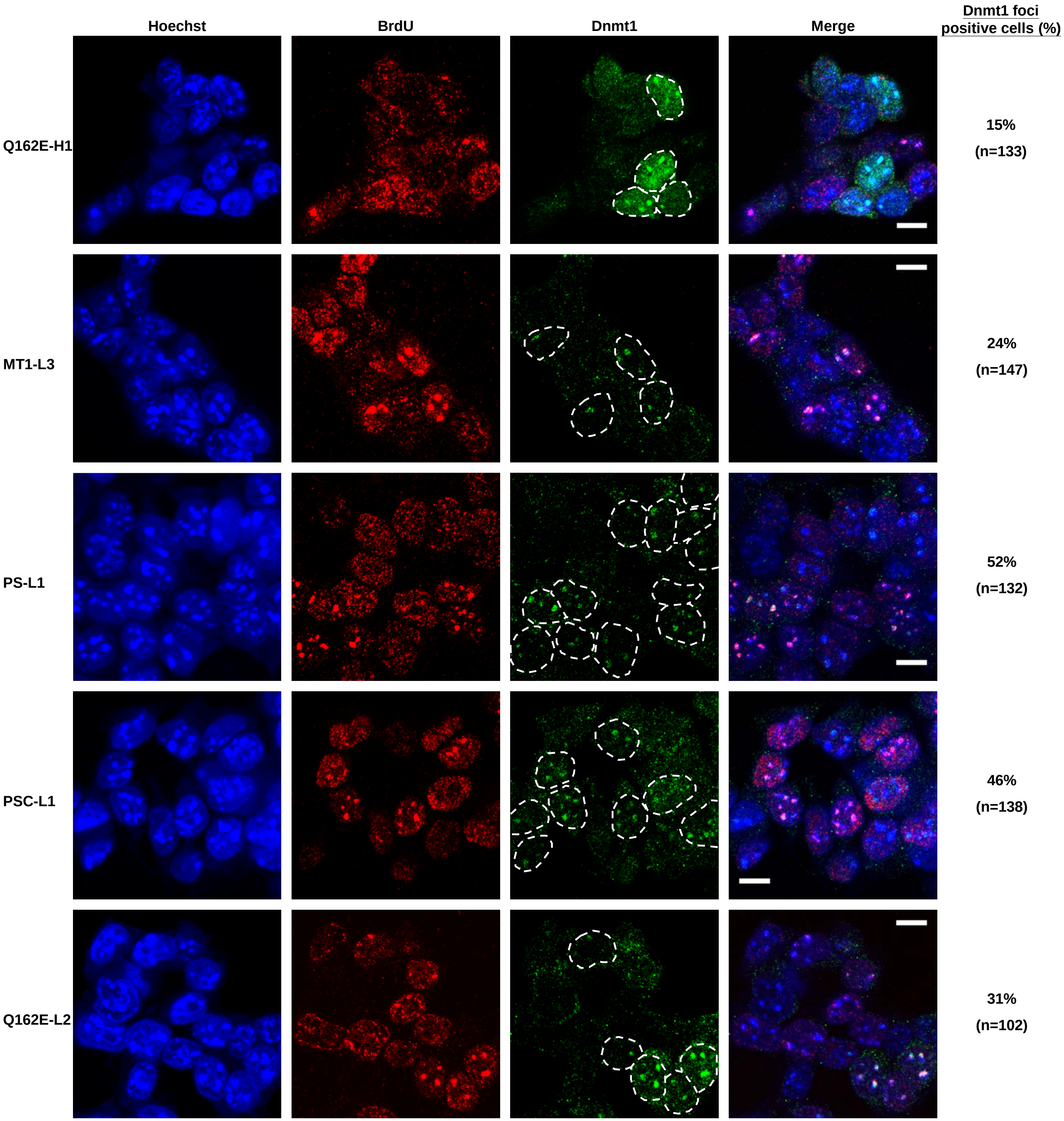

Supplementary Figure S2.

Enriched chromatin accumulation of the inactive Dnmt1 variants (PS and PSC)

As shown, 15~30% of Q162E-H1, MT1-L3 and Q162E-L2 cells contain Dnmt1 foci (indicated by the dashed circles, green) coinciding with the dense chromatin (Hoechst staining, blue). In PS-L1 and PSC-L1 cells, however, this percentage was increased to 46~52%. BrdU labeled the S phase cells (red).

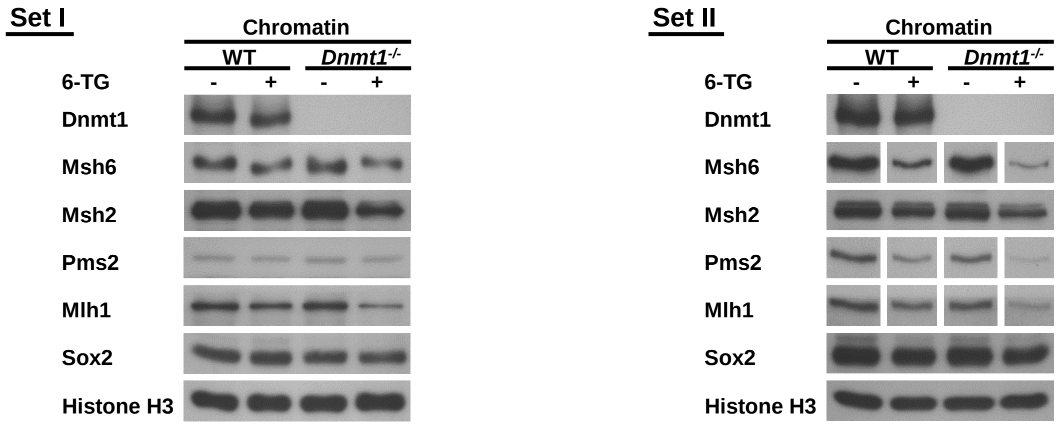

Supplementary Figure S3.

The replicated sets of Western blot images for the quantitative analysis of Fig. 4B

Western blotting patterns of the levels of different MMR proteins in the chromatin fractions of WT and *Dnmt1*$^{-/-}$ ES cells with or without 6-TG treatment are shown for replicated sets (I and II) of experiments. Histone H3 was used as the loading control of the chromatin fractions.

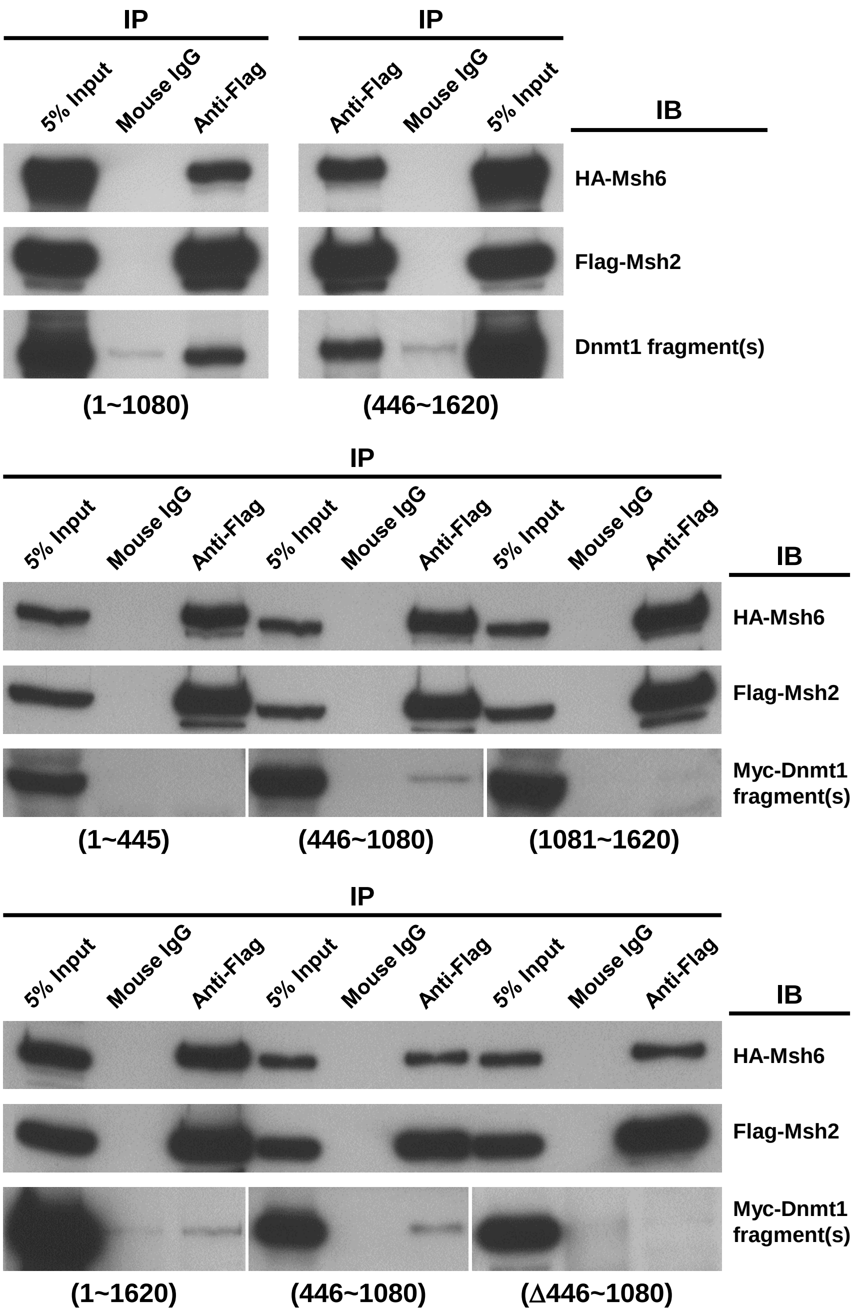

Supplementary Figure S4.

Mapping of the MutSα-interacting domains of Dnmt1

Co-IP assays of extracts from transfected 293T cells co-expressing different Dnmt1 fragments (with or without Myc-tag) with HA-Msh6 and Flag-Msh2 were performed to identify the domain(s) of Dnmt1 interacting with MutSα. Anti-Flag antibody was used for IP and mouse IgG was used as the negative control antibody. Anti-Flag, anti-HA, anti-Myc, or anti-Dnmt1 was used for IB. The mapping results from this set of IP/IB experiments are summarized in Fig. 5B.

Supplementary Table S1. **DNA oligo sequences**

Name	(Modification)Sequence	Applications	References
JH101F	(Hex)GTGGTAGAATACTTGGCTAAC	PCR of microsatellite marker JH101	1
JH101R	GCAGACTTGAGACTGTACTTG		
JH102F	(Hex)CATTTCTCTGGGATCGCCTT	PCR of microsatellite marker JH102	
JH102R	CCCGCCTTTGATTCCTTTGT		
D1Mit36F	GAGGAATGTAGAGTCCAACCTGG	PCR of microsatellite marker D1Mit36	2
D1Mit36R	(Hex)TGAATAGATTAAGAGCCTGGAAGC		
D7Mit91F	TCTTGCTTGCATACACTCACG	PCR of microsatellite marker D7Mit91	
D7Mit91R	(Fam)GAGACAAACCGCAGTCTCCT		
D14Mit15F	(Fam)TTGGCTGCTCACTTGCAG	PCR of microsatellite marker D14Mit15	
D14Mit15R	TTACCCTCCCCATAACTCCC		
Np95-gRNA-F	caccGCTTGCAGCTATCCAGAGCA	Construction of the Np95-gRNA expression plasmid	This study
Np95-gRNA-R	aaacTGCTCTGGATAGCTGCAAGC		
Np95-F	AGGGTGCTTGCTGAGCAGAG	PCR across the Cas9/gRNA target site of *Np95*	This study
Np95-R	GTATAGCTCACGTGCCGTCC		
Dnmt3a-QF	ATGTGGTTCGGAGATGGCAAG	Quantitative PCR of Dnmt3a	This study
Dnmt3a-QR	AGATGGCTTTGCGGTACATGG		
Dnmt3b-QF	GTTAATGGGAACTTCAGTGACCA	Quantitative PCR of Dnmt3b	3
Dnmt3b-QR	CTGCGTGTAATTCAGAAGGCT		

Supplementary References

1. Edelmann, W. *et al.* Mutation in the mismatch repair gene Msh6 causes cancer susceptibility. *Cell* **91**, 467-477 (1997).
2. Dietrich, W. F. *et al.* A genetic map of the mouse with 4,006 simple sequence length polymorphisms. *Nat. Genet.* **7**, 220-245 (1994).
3. Chen, T., Ueda, Y., Xie, S. & Li, E. A novel Dnmt3a isoform produced from an alternative promoter localizes to euchromatin and its expression correlates with active de novo methylation. *J. Biol. Chem.* **277**, 38746-38754 (2002).
